# Leukocyte profiles reveal sex and age differences in immune investment in a polygynous bat

**DOI:** 10.1111/1365-2656.70296

**Published:** 2026-06-30

**Authors:** Gerald S. Wilkinson, Jillian A. Kaiser, Katherine N. Armenta, Ana S. G. Cunningham, Severine B. S. W. Hex, Alexis M. Lawson, Jack G. Rayner

**Affiliations:** ^1^ Department of Biology University of Maryland College Park Maryland USA; ^2^ National Institute of Allergy and Infectious Diseases National Institutes of Health Bethesda Maryland USA; ^3^ School of Medicine University of North Carolina Chapel Hill North Carolina USA; ^4^ School of Psychology, Center for Research in Animal Behavior Exeter University Exeter UK

**Keywords:** cortisol, differential white blood cell counts, greater spear‐nosed bat, immunity life‐history trade‐off, neutrophil‐lymphocyte ratio, polygyny

## Abstract

1. Developing and maintaining an immune system is critical for vertebrate survival, but when resources are limiting, investment in support of immune function has been hypothesized to trade off with the development and/or expression of traits that enhance reproductive success. Species that exhibit extreme polygyny typically have high reproductive skew, which can be expected to result in sex differences in immune investment particularly for those individuals that succeed in reproducing. Furthermore, life history theory predicts that investment in adaptive immunity should decline as an individual approaches maximum lifespan and result in immunosenescence.
2. In this study, we evaluated these predictions by using differential white blood cell counts to assess relative investment in innate versus adaptive immunity over the lifespan of a sexually dimorphic neotropical bat that exhibits extreme polygyny. We also consider whether physiological stress or nutritional condition explains variation in leucocyte profiles.
3. We captured and made blood smears from 511 greater spear‐nosed bats, *Phyllostomus hastatus*, during periods of mating and lactation over 5 years and used a DNA methylation clock, recaptures of animals previously marked at a known age or sex‐specific regressions of toothwear on age to estimate age. We measured cortisol concentration from urine samples using gas chromatography, tandem mass spectrometry or an enzyme‐linked immunosorbent assay to measure physiological stress and sex‐specific residuals of body mass relative to forearm length to measure nutritional state.
4. We found that variation in the neutrophil‐to‐lymphocyte ratio (NLR) is associated with interactions between sex, age and season. NLR was higher in males than in females in all seasons and increased with age in both sexes, with the slope of age‐associated variation steeper in males than in females. Both cross‐sectional and longitudinal data revealed that NLR was highest in the mating season. Neither urinary cortisol nor body condition explained NLR variation within seasons.
5. Our findings provide evidence from a long‐lived bat that investment in innate immunity increases while adaptive immunity declines over the lifespan. Strikingly, this relationship occurs more rapidly in males than in females consistent with the higher mortality rate observed in males than in females of this highly polygynous species.

## Graphical abstract

Analysis of over 500 blood cell profiles, including repeat samples over four years, reveals that male bats from a long‐lived species with a polygynous mating system invest more in innate than adaptive immunity. Investment in innate immunity increases with age, as estimated by a DNA methylation clock and mark‐recapture data.

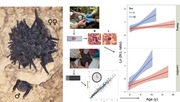

## 1 INTRODUCTION

The ability to resist or combat infection by pathogens, that is mount an effective immune response, is a major factor determining survival. However, developing and maintaining an immune system is costly (Lochmiller & Deerenberg, 2000). As a consequence, investment in immune function should trade off with investment in life‐history traits (Lee, 2006; Rauw, 2012; Rolff, 2002; Stoehr & Kokko, 2006; Zuk & Stoehr, 2002). Moreover, sex differences in reproductive effort or survival can be expected to cause trade‐offs involving immunity to differ between the sexes. For example, female mammals typically live longer than males (Lemaître et al., 2020; Promislow, 1992; Staerk et al., 2025). Several authors have proposed that in such species, males tend to invest less than females in immune defences because increased allocation of resources to sexually selected traits in males, such as ornaments, weapons or aggressive behaviours, can increase mating success and outweigh the costs of reduced lifespan (Lee, 2006; Zuk & Stoehr, 2002).

While it is commonly asserted that immune responses are lower in males than in females in many species (Klein & Flanagan, 2016; Nunn et al., 2009), a recent meta‐analysis that included data from over 100 invertebrate and vertebrate species failed to find evidence of an overall sex difference in immune function after controlling for effects of phylogeny (Kelly et al., 2018). However, this analysis did not consider the possibility that sex differences in immunity may vary among individuals depending on the mating system. In vertebrates, sex differences in immunity are expected to be greater for species with polygynous than monogamous mating systems (Klein, 2000b; Zuk & Stoehr, 2002), especially if the hormones that enhance male mating success, for example androgens, suppress immune function (Folstad & Karter, 1992; Klein, 2000a). While an early meta‐analysis of studies manipulating testosterone and measuring immune function failed to find evidence that testosterone impairs immune function (Roberts et al., 2004), a larger more recent meta‐analysis found that testosterone does have significant immunosuppressive effects (Foo et al., 2017).

Investment in each component of the vertebrate immune system, either the nonspecific innate or the specific acquired (adaptive), can be expected to be influenced by nutrition, pathogen exposure and extrinsic mortality risk. In particular, poor nutrition, low pathogen exposure and high mortality risk should favour investment in innate immune defences, while the opposite conditions should favour investment in adaptive immune defences (McDade et al., 2016). Evidence consistent with trade‐offs between immune components comes from artificial selection for reduced innate immunity in stickleback fish (*Gasterosteus aculeatus*), which resulted in upregulation of adaptive immune genes (Wegner et al., 2007). In addition, comparative studies in birds found that species with greater investment in adaptive immunity exhibit downregulation of their innate immune system (Minias et al., 2023).

As key constitutive components of the innate vertebrate immune system, circulating neutrophils (or heterophils in birds and reptiles) are rapidly recruited to destroy bacteria and fungi by phagocytosis, granule release and formation of extracellular traps (Papayannopoulos, 2018; Rosales, 2018). Neutrophils rarely survive in the blood for more than a day (Lahoz‐Beneytez et al., 2016), but proliferate in response to infections and physiological stress (Malech et al., 2014). In contrast, adaptive lymphocytes, consisting of B and T cells, modulate defence against previously encountered pathogens by regulating antibody production and quickly producing cytotoxins. While some lymphocytes may only survive a few days, others can survive for years. Given that neutrophils and lymphocytes typically comprise over 90% of circulating leukocytes, the ratio of neutrophils to lymphocytes, or NLR, provides a measure of the relative cellular investment in the non‐specific innate to the pathogen‐specific adaptive immune system.

Elevated NLR is used as a clinical biomarker of inflammation in humans (Garcia‐Escobar et al., 2023; Paganelli & Di Iorio, 2025) and as a measure of physiological stress in wild animals because elevated glucocorticoid levels can increase the number and percentage of neutrophils and decrease the number and percentage of lymphocytes (Davis et al., 2008; Gormally & Romero, 2020). In humans, NLR tends to be higher in males than females (Lin et al., 2016; Pellegrino et al., 2023), increases with age (Li et al., 2015; Pellegrino et al., 2024), and has been associated with mortality risk (Song et al., 2021). However, these patterns vary across taxa. NLR was higher in captive male chimpanzees (*Pan troglodytes*) but did not change with age over a 10‐year period (Webb et al., 2020), while in captive baboons (*Papio anubis*), NLR was higher in females and increased until mid‐life before decreasing with age (Neal et al., 2024). In contrast, in bigmouth buffalo (*Ictiobus cyprinellus*), an extremely long‐lived freshwater fish, NLR decreased with age in both sexes (Sauer et al., 2021). In a comparative study of birds, the heterophil‐to‐lymphocyte ratio correlated negatively with longevity (Minias, 2019) consistent with long‐lived species investing more in adaptive than innate immunity.

In this study, we aimed to determine whether variation in NLR is associated with sex, age, nutritional status or physiological stress in an extremely polygynous mammal, the greater spear‐nosed bat, *Phyllostomus hastatus*. In this species a dominant ‘harem’ male aggressively defends and mates with 15–25 females in a roosting group for up to 4 years (Adams & Wilkinson, 2020; McCracken & Bradbury, 1981). Female group membership is often stable with some individuals remaining in the same group for over 10 years (Wilkinson et al., 2016; Wilkinson et al., 2019). In the wild, females have lower mortality rates than males and can live nearly twice as long—up to 22 years of age (Adams et al., 2025). Harem males actively defend females year‐round (McCracken & Bradbury, 1981) and are typically in better body condition, that is higher mass relative to body size, than non‐harem ‘bachelor’ males (Adams & Wilkinson, 2020). Harem males also have lower mortality rates and tend to be older than bachelor males (Adams et al., 2025). The differences in survival between the sexes and male statuses are reflected in patterns of DNA methylation, with higher mortality rates associated with greater rates of epigenetic change (Adams et al., 2025).

Given the mating system and differences in mortality between the sexes and male statuses, we predicted that males would have higher NLR than females due to higher investment in innate than adaptive immunity and that both sexes would exhibit increased NLR with age due to senescence of adaptive immunity, possibly with this effect being more pronounced in bachelor than harem males. Furthermore, if elevated NLR is indicative of physiological stress, we predicted that NLR would be positively related to urinary cortisol levels (Davis et al., 2008). NLR could also be influenced by nutritional stress (Carbillet et al., 2019), in which case we predicted that it would be negatively related to body condition. In this species, body condition changes over the year with females having lower mass during the mating period than during the lactation period while body condition of males is positively associated with age in the mating period but not during the lactation period (Wilkinson et al., 2024). Thus, we also evaluate the effect of season on NLR and consider the possibility that there could be seasonal differences in NLR between males and females.

## 2 METHODS

Greater spear‐nosed bats, *Phyllostomus hastatus*, were captured in five cave or building roosts in Trinidad, Lesser Antilles, using a bucket trap or hand net before transfer to a cloth bag. Roost sites sampled include three caves (Caura, Colado and Guanapo) in the northern range, Tamana cave, in the central range and an abandoned cold storage building in the town of Cumuto. This study was enabled by prior studies at these sites (e.g. Adams et al., 2025; Wilkinson, 2025; Wilkinson et al., 2024), which made it possible to determine ages of many individuals from recaptures. Bats were captured and sampled during the mating season in January of 2023, 2024, 2025 and 2026, the lactation period in May of 2023, and the post‐lactation period in July of 2011, with the older samples collected as part of a previous study (Adams & Wilkinson, 2020). One of the sites visited in 2011 was destroyed in 2012, which is why it was not visited again. In Trinidad, mating occurs between November and January (McCracken & Bradbury, 1981) at the beginning of the dry season when the bats are feeding primarily on pollen and fruit (Wilkinson & Boughman, 1998). Starting in their second year, females give birth to a single pup in April or May (Porter & Wilkinson, 2001), which coincides with the start of the rainy season when the bats consume much more insect material (Wilkinson & Boughman, 1998).

Upon capture, bats were sexed, forearm length was measured to 0.01 mm, mass was weighed to the nearest 0.5 g, and toothwear was scored on a 10‐point scale, where a score of 1 indicates no discernable wear and a score of 10 indicates lack of canines and extremely worn molars, with intermediate scores between (Adams & Wilkinson, 2020). Individuals not previously captured were marked with a metal alloy band stamped with an identification number. Male status, harem or bachelor, was determined based on whether the male was captured while defending a group of females or roosting predominantly with other males, respectively. Two males initially captured as bachelor males were subsequently captured several years later as harem males, indicating they had gained control of a female group. One or two 4‐mm biopsy punches were taken from the wing, placed in Zymo DNA/RNA Shield (ZymoResearch, Orange, CA, USA) in the field, and then stored at −20°C.

To obtain blood for smears, we punctured the antebrachial vein using a sterile lancet and then used a heparinized capillary tube to collect 2–3 μL of blood to make a smear. Blood smears were made for a total of 511 individuals (226 males and 285 females, see Table S1 for counts by season, site and year). We captured animals during the day with a median collection time of 13:04 (interquartile range: 11:40–13:35). Animals were released into their roost within 3 h of capture. Smears were air‐dried and then fixed with methanol within 24 h. Fixed smears were subsequently stained (Hema 3, Fisher Scientific) in the laboratory and then scored blind by at least three people who had been trained to distinguish cell types. Each person scanned the smear at 200x using a Nikon Eclipse E600 microscope and identified at least 100 leukocytes as either neutrophil, lymphocyte, monocyte, eosinophil or basophil. Any slide that had scores resulting in a coefficient of variation (CV) for percent neutrophil of over 30% was either rescored or, if the original scorer was unavailable, the outlier score was excluded (*N* = 18). Across the entire data set, which includes replicate slides for some individuals, neutrophil CV = 10.24 ± 0.27% (*N* = 582), indicating consistency across scorers.

We determined age in one of three ways. In order of priority they were (1) recaptures of known‐age animals, *N* = 154, (2) predictions derived from an epigenetic clock, *N* = 215 (Wilkinson et al., 2021) or (3) predictions derived from toothwear scored on a 10‐point scale, *N* = 141 (Adams et al., 2025). To obtain the epigenetic clock estimate, DNA was extracted from wing punches using a Zymo Quick‐DNA miniprep plus kit using tissue protocols. Samples with a DNA concentration below 10 ng/μl were concentrated using a 30 kDa centrifugal filter (MilliporeSigma, Burlington, MA, USA) until they had at least 12 ng/μL in 20 μL. Methylation was measured by the Clock Foundation (Torrance, CA, USA). After bisulphite conversion, samples were hybridized to a custom Illumina microarray (Arneson et al., 2022). The proportion of DNA molecules methylated at each CpG site (i.e. Beta values) were obtained after normalization using the SeSaME procedure (Zhou, Triche, Laird, & Shen, 2018). Based on prior comparison of age estimates using several different DNA methylation clocks, we used the estimates provided by the ‘AllBat’ clock, as this clock provided the most accurate prediction of intervals between successive captures of the same animal (Adams et al., 2025). In addition, chronological age was highly predictive of the clock age estimate (*r* ^2^ = 0.923, intercept = 0.09 ± 0.34, slope = 1.13 ± 0.06) for 34 animals for which both estimates were available. Age estimates based on toothwear were not as accurate (Figure S1) but still explained more than 60% of the variation in age (males: *n* = 135, *r* ^2^ = 0.611, intercept = −1.383 ± 0.395, slope = 2.617 ± 0.181; females: *n* = 478, *r* ^2^ = 0.677, intercept = −2.427 ± 0.311, slope = 3.495 ± 0.111). Distributions of age estimates (Figure S2) revealed that the oldest female sampled was 19.8 years of age while the oldest harem male was 12.6 and the oldest bachelor male was 9.3 years of age.

We used the neutrophil‐to‐lymphocyte ratio (NLR) as an estimate of the relative investment in constitutive innate versus adaptive immunity. The distribution of NLR was strongly right‐skewed (Pearson's median skew = 0.8187), so we used the natural logarithm (ln) to transform NLR, which then closely approximated a normal distribution (mean = 0.359, standard deviation = 0.870, skew = 0.0534). Consequently, ln(NLR) >0 indicates more neutrophils than lymphocytes, whereas ln(NLR) <0 indicates more lymphocytes than neutrophils. We did not analyse variation in monocytes, eosinophils or basophils as they typically constituted less than 10%, on average, of identified white blood cells, and preliminary analyses failed to detect any changes with sex or age.

NLR could potentially be influenced by nutritional state or physiological stress. To assess nutritional state, we measured body condition as the residual of body mass regressed on forearm length by sex. Forearm length is a very reliable measure of skeletal size in a bat. For example, for the 146 animals captured and measured in two or more years, average CV for forearm length was 0.31 ± 0.04%. We measured condition for all animals sampled for NLR, but we excluded 14 juveniles captured during the lactation period because they were not fully grown and four adults that were captured after they had returned from feeding. We estimated residuals from a model that controlled for sex because males are 20% heavier than females (Adams & Wilkinson, 2020).

To assess physiological stress, we estimated urinary cortisol concentration. We used cortisol because it is the biologically active glucocorticoid in bats (Widmaier & Kunz, 1993) and was the most abundant glucocorticoid metabolite present in urine (Wilkinson et al., 2024). We estimated cortisol levels in urine collected from 76 female and 70 male adult bats during the mating or lactation periods (Table S2). To collect urine, individuals were placed in a modified plastic bucket with a funnel bottom fitted with a removable 5‐mL collection tube beneath fine mesh to exclude faecal material. Bats were left undisturbed in buckets for 1–3 h before tubes were removed, placed on ice and frozen at −20°C within 5 h of collection. For samples with sufficient volumes, we soaked Whatman 903 filter paper strips with 500 μL of urine, dried and then stored them with silica gel at −20°C until processing. Glucocorticoids (cortisol, cortisone, tetrahydrocortisol and tetrahydrocortisone) were measured from dried urine by ZRT Labs using gas chromatography‐triple quadrupole mass spectrometry (GC–MS/MS). Samples were extracted from filter paper strips, and a 2 μL volume of product was injected into an Agilent 7890 Triple Quadrupole Mass Spectrometer. Results were analysed using the MassHunter software, v.12. We corrected for variation in the water content of urine by measuring and then adjusting for specific gravity (SG). We measured SG at room temperature using an optical refractometer (Aichose) with automatic temperature compensation. Following Miller et al. (2004), we multiplied the hormone metabolite estimate of individual *x* by the following correction factor: (mean SG − 1)/(SG(*x*) − 1). We used the natural logarithm of cortisol concentration, after adjusting for specific gravity, so the distribution would approximate normality. Finally, because urinary cortisol concentration changes throughout the day, we used the residuals from a regression of ln(cortisol‐SG) on collection hour (Wilkinson et al., 2024).

To identify variables that explain variation in NLR, we fit a series of linear mixed models. We first compared models that included sex, age, season and all possible interactions, with individual included as a random effect to account for measurements taken from the same individual at more than one time point. To determine whether NLR differed between bachelor and harem males, we fit models with age, status and their interaction as fixed effects and season and individual included as random effects. We chose models for interpretation by minimizing the corrected Akaike information criterion (AICc). If models were functionally equivalent, that is ΔAICc <2, we chose the simplest model to avoid including uninformative parameters (Leroux, 2019; Sutherland et al., 2023). To determine significance of differences among states of categorical variables, we conducted Tukey honestly significant difference (HSD) post hoc tests on all pairwise comparisons.

To test whether variance in NLR decreased with age, as would be expected if mortality was associated with either consistently high or low NLR, which would reduce variation in NLR among older individuals, we conducted Breusch–Pagan (BP) tests by calculating the squared residuals from the selected model and then used those residuals in a regression against age for each sex. We calculated the chi‐squared test statistic as n*R ^2^ to evaluate significance (Breusch & Pagan, 1979).

In addition, to cross‐sectional analyses, we also conducted longitudinal analyses to determine whether seasonal differences in NLR could be detected within individuals. We used matched pairs *t*‐tests to compare NLR for 40 animals (23 females, 17 males) captured and sampled in the lactation period and at least one mating period. Given that we only sampled animals in one lactation period, it is possible that annual variation might contribute to any seasonal difference. Therefore, to determine whether there were significant differences across years for animals caught in the same season, we included year as a fixed effect in a linear mixed model for NLR, with individual included as a random effect. For this analysis, we only included animals that were sampled in more than one mating season from 2 to 4 years. We also used matched pairs *t*‐tests to compare NLR for individuals sampled in consecutive years. Data from 2026 were added after initial submission, and their inclusion increased our ability to quantify longitudinal change.

In a prior study, body mass was reported to be lower and cortisol levels were reported to be higher during the mating season (Wilkinson et al., 2024). Therefore, to determine whether body condition or cortisol concentration covaried with NLR within a season, we used each of those variables in linear mixed models that also included sex, age and their interaction with individual and season as random effects. Because our intent was to test whether either variable explained variation in NLR beyond that of sex, age and season, we report the best‐fitting models that included these variables and then use parameter estimates from those models to indicate whether condition or cortisol covary with NLR and thereby indicate if NLR is associated with nutritional state or physiological stress.

All statistical tests were conducted using JMP 19.0.1. (SAS Institute Inc, 2026).

### 2.1 Ethics approval

Animal handling methods follow guidelines by the American Society of Mammalogists and were approved by the University's Institutional Animal Care and Use Committee (protocols FR‐JUL‐24‐25, FR‐JUL‐21‐46, FR‐APR‐18‐16) under licences from the Forestry Division of the Ministry of Agriculture, Land and Fisheries, Trinidad and Tobago.

## 3 RESULTS

### 3.1 NLR is influenced by sex, age and season

**FIGURE 1:**
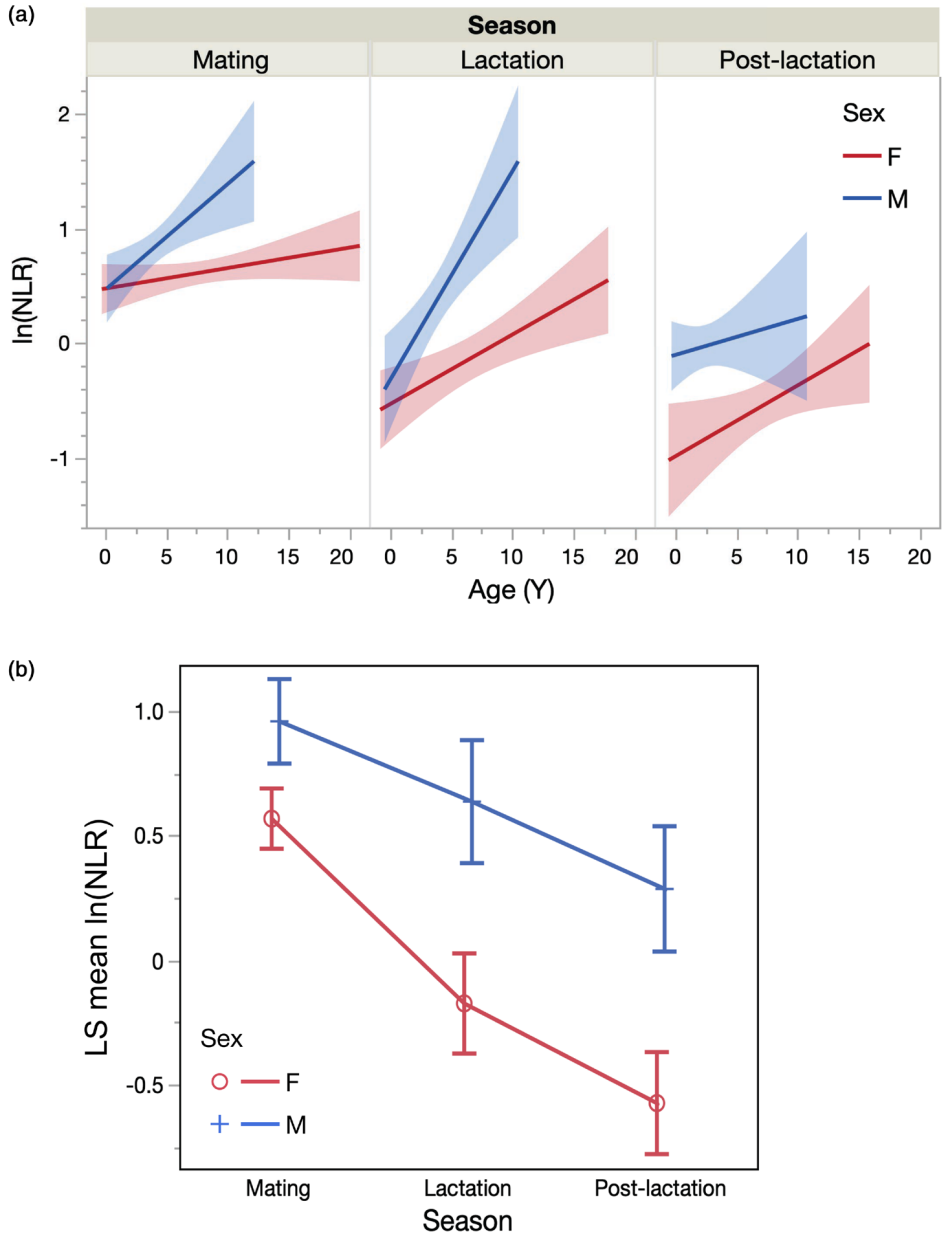
Plots of (a) predicted ln(NLR) with 95% confidence intervals by age for each sex with females in red and males in blue and (b) least squares mean ln(NLR) by season for each sex with 95% confidence intervals.

Comparison of mixed models for NLR using sex, age and season as fixed effects and individual as a random effect reveals that a full model with all possible interactions had the best fit but a model with only two‐way interaction terms was functionally equivalent, that is ΔAICc = 0.9 (Table 1). Examination of the parameter estimates for the simpler model (Table 2) indicates that NLR was positively associated with age, differed by sex and season, and was influenced by several interactions (Figure 1a). Tukey's post hoc comparisons reveal that male NLR was higher than female NLR (M‐F = 0.687 ± 0.097, *t* = 7.01, *p* < 0.0001, Figure 1b) and that NLR was higher during the mating season than during the lactation or post‐lactation periods (ln(NLR)_M‐L_ = 0.533 ± 0.084, *t* = 6.35, *p* < 0.0001; ln(NLR)_M‐PL_ = 0.908 ± 0.083, *t* = 11.01, *p* < 0.0001; ln(NLR)_L‐PL_ = 0.376 ± 0.102, *t* = 3.68, *p* < 0.0008). Post hoc comparison of the NLR–age relationships between the sexes reveals that the slope was steeper in males than females (difference = 0.058 ± 0.025, *t* = 2.27, *p* = 0.0237). In contrast, post hoc comparisons of the NLR‐age slopes between the seasons indicate that only lactation and mating slopes differ, with the lactation slope being steeper (difference = 0.053 ± 0.021, *t* = 2.55, *p* = 0.0297). The variation ascribed to individual in the model was significant, that is the intraclass coefficient or repeatability was 0.317 (Wald *p* = 0.0007).

**TABLE 1:** Comparison of fit as measured by the corrected Akaike Information Criterion (AICc) of linear mixed models that predict ln(NLR) using season, sex, age and interactions as fixed effects and individual as a random effect (*N* = 511).

Model	AICc	ΔAICc
Season*Sex*Age	1120.54	0.00
Season + Sex + Age + Sex*Season + Sex*Age + Season*Age	**1121.45**	**0.91**
Season + Sex + Age + Sex*Season + Sex*Age	1123.83	3.29
Season + Sex + Age + Age*Season + Sex*Season	1124.59	4.05
Season + Sex + Age	1125.58	5.04
Season + Sex + Age + Age*Sex	1127.08	6.54
Season + Sex + Age + Sex*Season	1127.52	6.98
Season + Sex + Age + Age*Season	1128.35	7.81
Season + Sex + Sex*Season	1148.16	27.62
Season + Sex	1148.63	28.09

*Note*: Parameter estimates for bold model in Table 2.

**TABLE 2:** Parameter estimates for the linear mixed model that predicts ln(NLR) as indicated in Table 1.

Term	Estimate	SE	df	*t*	*p*
Intercept	−0.138	0.069	472.8	−2.00	0.0460
Sex[F]	−0.344	0.048	415.7	−7.10	< 0.0001
Age	0.071	0.013	442.9	5.45	< 0.0001
Season[M]	0.480	0.044	463.7	10.96	< 0.0001
Season[L]	−0.052	0.056	426.8	−0.94	0.3491
Sex[F]*(Age − 5.94)	−0.029	0.013	409.4	−2.27	0.0237
Season[M]*Sex[F]	0.148	0.050	477.6	2.97	0.0032
Season[L]*Sex[F]	−0.062	0.061	454	−1.01	0.3110
Season[M]*(Age − 5.94)	−0.022	0.012	480.9	−1.81	0.0703
Season[L]*(Age − 5.94)	0.031	0.015	460	2.12	0.0347

The association of NLR with age is not likely due to differential mortality because variation in NLR did not change with age: a BP test using squared residuals from the NLR model fit to age was not significant (*R* ^2^ = 0.0013, *n* = 511, *χ* ^2^ = 0.654, *p* = 0.419). BP tests using squared residuals from NLR models fit to age separately for each sex were also not significant (females: *R* ^2^ = 0.0119, *n* = 285, *χ* ^2^ = 3.401, *p* = 0.065; males: *R* ^2^ = 0.0057, *n* = 225, *χ* ^2^ = 1.289, *p* = 0.256). While the female BP test approached significance, variation in NLR showed a trend to increase, rather than decrease, with age as expected if changes in NLR were associated with differential mortality.

Among males, NLR was not influenced by status beyond an effect of age. Including male status or the interaction between male status and age as fixed effects with individual and season as random effects did not improve fit over a model that only contained age (Table S2).

**FIGURE 2:**
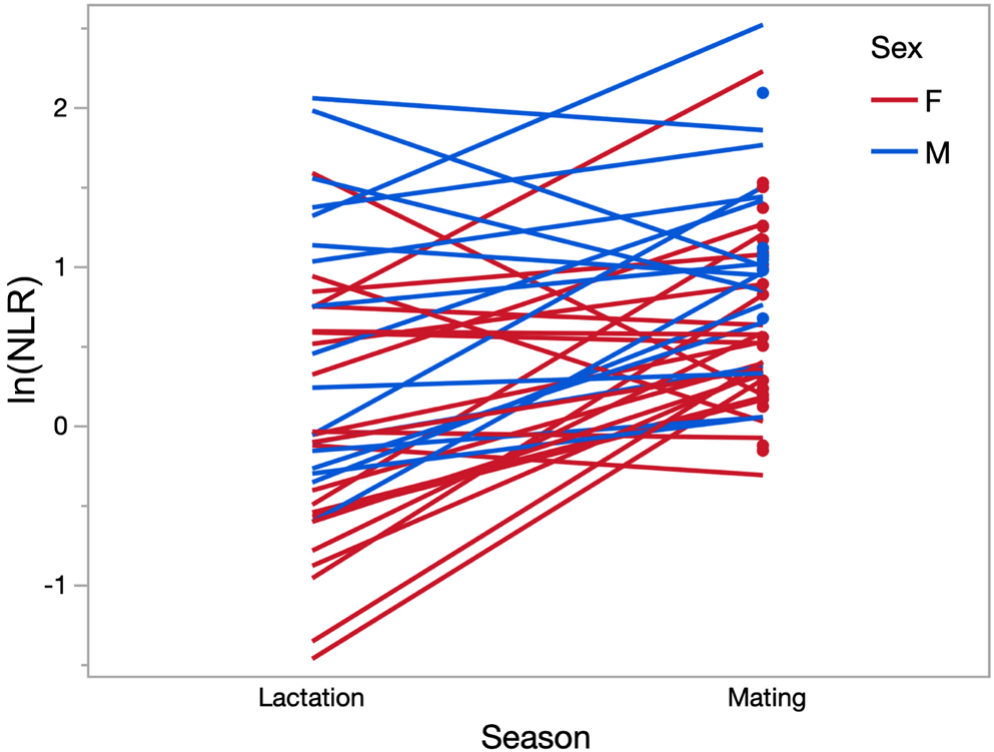
Neutrophil‐to‐lymphocyte ratio (lnNLR) for 40 individuals sampled both in the mating and lactation periods, with females in red and males in blue.

Season also influenced NLR within individuals. Comparison of NLR across seasons for the same animal revealed no effect of sex but a significant effect of season (paired *t* = 4.27, *p* = 0.0001, DF = 39) with ln(NLR) elevated by 0.540 ± 0.126 during the mating period compared to the lactation period (Figure 2a). Because we only measured NLR during the lactation period in 1 year, a seasonal difference might be influenced by annual fluctuations. However, year was not significant in a mixed model for NLR that also included sex as a fixed factor and individual as a random effect (*F*_3,88.8_ = 0.87, *p* = 0.4606, Figure S3). Matched pair comparisons within individuals across successive years also revealed no effect of year (2023–24: *t* = 1.03, DF = 9, *p* = 0.33; 2024–25: *t* = 0.26, DF = 10, *p* = 0.80; 2025–26: *t* = 0.02, DF = 14, *p* = 0.98).

### 3.2 NLR and urinary cortisol

Urinary cortisol exhibited a similar seasonal pattern as NLR, with it being higher during the mating period than during the lactation period (Figure S4). To determine whether urinary cortisol concentration was associated with NLR within seasons, we included cortisol levels in a mixed model for NLR with sex, age and interactions as fixed effects and season and individual as random effects. Model comparison (Table 3) revealed that a model with just sex and age fit the data a bit better than a model that included cortisol, sex and age (ΔAICc = 1.4). Examination of parameter estimates for the three‐factor model (Table 4) showed that cortisol concentration did not explain enough variation for the coefficient to differ from zero.

**TABLE 3:** Comparison of fit as measured by the corrected Akaike Information Criterion (AICc) of linear mixed models that predict ln(NLR) using ln cortisol concentration (cort)—corrected for specific gravity and time of day, sex, age and interactions as fixed effects with individual and season as random effects (*N* = 146).

Model	AICc	ΔAICc
Sex + Age	293.68	0.00
**Cort + Sex + Age**	**295.16**	**1.48**
Sex*Age	295.91	2.22
Cort + Sex + Age + Sex*Cort	296.36	2.67
Cort + Sex + Age + Age*Cort	297.37	3.68
Cort + Sex + Age + Age*Sex	297.39	3.71
Cort + Sex + Age + Sex*Cort + Sex*Age	298.60	4.91
Cort + Sex + Age + Age*Cort + Sex*Cort	298.63	4.94
Cort + Sex + Age + Sex*Cort + Sex*Age + Cort*Age	300.91	7.22
Cort*Sex*Age	302.38	8.69

*Note*: Parameter estimates for bold model in Table 4.

**TABLE 4:** Parameter estimates for the linear mixed model that predicts ln(NLR) as indicated in Table 3.

Term	Estimate	SE	DF	*t*	*p*
Intercept	0.141	0.304	1.3	0.46	0.7107
Sex[F]	−0.229	0.066	116.8	−3.48	0.0007
Age	0.046	0.018	120.6	2.56	0.0117
Cortisol	−0.070	0.079	99.9	−0.88	0.3787

### 3.3 NLR and body condition

Similar to urinary cortisol, body condition differed between seasons (Figure S5). Thus, to determine whether elevated NLR was associated with nutritional stress, we included body condition in addition to sex, age and interactions in mixed models for NLR with season and individual as random effects. Model comparison reveals that an additive model for NLR that included only sex and age fit much better (ΔAIC = 10.63) than the best model that included condition (Table S4).

## 4 DISCUSSION

We found that male greater spear‐nosed bats had higher NLR than females and more circulating neutrophils than lymphocytes. In addition, NLR was highest during the mating season in both sexes. NLR was positively associated with age in both sexes, and this association was stronger in males. These data are consistent with increased investment in innate immunity with increasing age, while investment in adaptive immunity declines, especially in males. This pattern of immunosenescence is not uncommon. Age‐related declines in adaptive immune responses have been more commonly reported than for innate immune responses in 44 species of captive and free‐living mammals, birds and reptiles (Peters et al., 2019). In addition, a decline in adaptive immunity is well known in humans and explains why vaccine efficacy declines with age (De Maeyer & Chambers, 2021; Goronzy & Weyand, 2013).

While sex differences in immune responses are well‐characterized in humans (Klein & Flanagan, 2016; Sharma et al., 2025), evidence for consistent sex differences in immune responses in nonhuman animals is more equivocal. Despite suggestions that immune responses of females should be greater than for males due to life‐history differences (Lee, 2006), a meta‐analysis of over 100 studies failed to find consistent evidence for such an effect (Kelly et al., 2018). One possible explanation for the observed variation is that species with diverse mating systems have been considered together. For example, of the 14 species of mammals included in the Kelly et al. (2018) study, 10 were rodents and only one species could be considered polygynous. Greater spear‐nosed bats exhibit extreme polygyny in which one male can potentially sire 80 or more offspring during his tenure as a harem male (Adams & Wilkinson, 2020; McCracken & Bradbury, 1981). Bayesian analysis of mark–recapture data indicates that males have much higher mortality rates than females, and bachelor males have higher mortality rates than harem males (Adams et al., 2025). Interestingly, mortality rates of male greater spear‐nosed bats are also higher than females in captivity (Staerk et al., 2025). Thus, to the extent that NLR indicates constitutive investment in innate versus adaptive immunity, these results are consistent with the sex with the higher rate of mortality investing more in innate than adaptive immunity. These results are consistent with comparative studies in birds, which show that species with greater maximum lifespan tend to have lower HLR (Minias, 2019) and with a study on roe deer (*Capreolus capreolus*), which found that males exhibited an increase in NLR with age that was greater than observed in females in two populations (Carbillet et al., 2019).

Sex differences in immunity can be caused by sex differences in pathogen exposure. Differences in pathogen exposure are unlikely to explain the differences in NLR we observed because both males and females spend the day roosting in an enclosed communal site, such as a cave, and are often in close proximity to each other. While the bats' diet changes over the year (Wilkinson & Boughman, 1998), there is no evidence that males consume different food items than females. We did not observe a sex difference in the occurrence of any blood parasites, which were very rare, in the blood smears. However, males may be more prone to inflammation caused by injuries. Males engage in aggressive interactions that often involve biting that can result in torn ears, broken canines and obvious teeth mark punctures on the wings and arms. While it is possible that NLR could change as a consequence of such interactions, infra‐red illuminated video recordings of males in roosts during the mating season indicate that intense, prolonged fights more commonly involve bachelor than harem males (unpublished data, S.B.S.W. Hex & G.S. Wilkinson) and consequently are more likely to influence NLR in bachelor males. Given that harem males are typically older and in better body condition than bachelor males (Adams et al., 2025; Adams & Wilkinson, 2020), and yet NLR is positively associated with age in both sexes, investment in adaptive immunity apparently declines as an individual of either sex approaches its maximum lifespan.

We detected substantial seasonal differences in NLR in both cross‐sectional and longitudinal data. The fact that NLR is higher during the mating season when cortisol levels are higher and body condition is lower (Wilkinson et al., 2024) suggests either that glucocorticoid levels, food availability or both could be contributing factors. Females have elevated levels of most steroid hormone metabolites in their urine during the mating season. In contrast, male steroid hormone metabolites are influenced more by age than by season (Wilkinson et al., 2024), with older males excreting high levels of androgens and glucocorticoids in all seasons. During the mating period, greater spear‐nosed bats feed predominantly on pollen, nectar or fruit, which likely provide energy but not much protein. In contrast, the bats consume more insect material during the lactation period, which likely explains the seasonal differences in condition (Wilkinson, 2025). Interestingly, NLR was also higher in wild roe deer (*Capreolus capreolus*) during stressful years when fawn mass was low (Carbillet et al., 2019).

Given the seasonal associations between NLR, cortisol and body condition, we predicted that elevated NLR would be positively associated with urinary cortisol concentration and negatively associated with condition, as measured by sex‐specific residual body mass. However, after controlling for sex, age and season we failed to detect any significant associations. Thus, these data suggest that NLR is not related to condition or physiological stress. However, it is important to recognize that leukocyte counts, urinary hormone levels and body mass measurements are the result of physiological processes that involve different tissues and responses that occur over different time scales (Davis & Maney, 2018). Comparison of biomarkers measured from the same tissue source might reveal different patterns. For example, glucocorticoid levels measured in plasma could differ from what we detected in urine and possibly be associated with NLR. Unfortunately, the process of taking a blood sample is stressful and can influence cortisol levels more quickly (Widmaier & Kunz, 1993) than NLR, which makes such comparisons difficult. In addition, chronic stress can eventually result in attenuation of a glucocorticoid response while NLR can remain high over longer periods (Davis & Maney, 2018). More studies that measure how NLR responds to acute and chronic stress in bats would be useful.

A potential criticism of this study is that much of the evidence for immunosenescence comes from cross‐sectional data, which can yield biased age effects if immune function covaries with survival. While our study did utilize cross‐sectional data, we tested for age‐related changes in NLR variation, which would be expected to decrease if there were differential mortality, and found no evidence that NLR variation changes with age. Furthermore, we did not sample bats at every site, season and year, so we cannot determine if NLR differs among some sites. Given that prior studies have reported small differences in the timing of parturition across the island (Porter & Wilkinson, 2001), some associated differences in NLR seem likely. Nevertheless, repeated measurements on 40 individuals sampled in multiple years revealed that the seasonal differences in NLR we observed in cross‐sectional data at several sites are also seen in longitudinal data for individuals of both sexes sampled at the same site. In addition, we did not detect significant differences associated with year for animals that were resampled during the mating period over a four‐year period at the same site. Thus, while it is possible that differences among sites could explain some additional variation in NLR, the consistency between the cross‐sectional and longitudinal seasonal analyses, as well as differences in NLR between the sexes observed at nearly all ages, provides some assurance that the sex and age differences in NLR are not aberrant or a consequence of differences in survival.

In this study, we focussed on comparing constitutive components of the immune system in free‐ranging bats. While we did not experimentally manipulate the animals to induce an immune response, the seasonal variation that we observed could be considered a consequence of a natural experiment, which altered stress. While urinary cortisol and body condition differ between the mating and lactation periods in directions that could influence NLR, neither factor explained variation in NLR within seasons. Experimentally inducing an immune response, either in vivo or ex vivo, would provide useful additional information on the extent to which immune responses differ between the sexes or with age. However, many of the methods that have been used to quantify immune responses in nonhuman animals (Kelly et al., 2018; Peters et al., 2019) were originally developed for biomedical purposes. Given that the genes involved in the immune systems of bats differ in a variety of ways from other mammals (Gorbunova et al., 2020; Moreno Santillan et al., 2021; Scheben et al., 2023; Zhang et al., 2013; Zhou et al., 2014; Zhou et al., 2016), rather than measure particular gene products, such as cytokines, we suspect it will be more informative to measure induced immune responses by quantifying transcriptomic or proteomic changes in an unbiased way.

## 5 CONCLUSIONS

The results of this study demonstrate that investment in innate versus adaptive immunity varies with sex and age in the highly polygynous greater spear‐nosed bat. In this species, NLR is elevated in males, in older individuals and during the mating season, collectively contributing to our understanding of how immune investment is shaped by a complex interaction of individual phenotype and environmental stressors. Further, by studying a species with extreme polygyny and male reproductive skew, we add to the literature of how mating system may be reflected in heterogeneous immune trade‐offs between individuals in the same population. Further work on species with diverse mating systems, as well as longitudinal studies following known individuals in the wild and ex vivo experiments, will allow us to gain a better understanding of how individuals balance reproductive effort with immune investment, and how this trade‐off shapes the evolution of life‐history strategies.

## AUTHOR CONTRIBUTIONS

Gerald Wilkinson conceived of the idea and designed methodology; Jillian Kaiser, Katherine Armenta, Ana Cunningham, Severine Hex, Alexis Lawson, Jack Rayner and Gerald Wilkinson collected data; Gerald Wilkinson and Jillian Kaiser analysed the data; Gerald Wilkinson led the writing of the manuscript. All authors contributed critically to drafts and gave final approval for publication.

## CONFLICT OF INTEREST STATEMENT

The authors declare no conflicts of interest.

## Supporting information


**Table S1.** Number of individual bats sampled for NLR and body condition by sex, season, site and year.
**Table S2.** Number of individual bats sampled for cortisol by sex, season, site and year.
**Table S3.** Comparison of fit as measured by the corrected Akaike Information Criterion (AICc) of linear mixed models that predict ln(NLR) in 209 adult males (147 bachelor, 62 harem) using status, age and their interaction as fixed effects and individual and season as random effects.
**Table S4.** Comparison of fit as measured by the corrected Akaike Information Criterion (AICc) of linear mixed models that predict ln(NLR) using sex‐specific relative body mass (condition), sex, age and interactions as fixed effects with individual and season as random effects (*N* = 494).
**Figure S1.** Relationship between age estimated by DNAm or mark recapture interval plotted against toothwear index for each sex. Least squared regression used to predict age for females is age = −2.0591 + 3.4318(TI), *r* ^2^ = 0.652, *N* = 520 and for males it is age = −1.2719 + 2.5798 (TI), *r* ^2^ = 0.604, *N* = 156.
**Figure S2.** Distribution of ages of wild female (F) and male (M) greater spear‐nosed bats with estimates of NLR. The method of age estimation is indicated as DNA methylation clock (DNAm), recapture of a bat whose age had previously been estimated with the DNA methylation clock (DNAmR), recapture of a bat initially banded as a pup (Recap), or using a sex‐specific regression derived from comparing known aged animals to their toothwear score (cf. Figure S1).
**Figure S3.** Neutrophil‐to‐lymphocyte ratio (lnNLR) for animals sampled during the mating season in more than 1 year with females in red and males in blue.
**Figure S4.** Plots of ln(cortisol corrected for specific gravity) and residual ln(cortisol corrected for specific gravity and collection hour) with median, quartiles and outliers by season with females in red and males in blue.
**Figure S5.** Plots showing median, quartiles and outliers for body condition (sex‐specific residual body mass relative to forearm length) by season with females in red and males in blue.

## Data Availability

Leukocyte proportions and cortisol data are available on figshare at https://doi.org/10.6084/m9.figshare.30471803 (Wilkinson, 2026). Methylation data are available from NCBI Gene Expression Omnibus under accessions GSE273259 and GSE331479.
