# Supplementary material for: Leukocyte profiles reveal sex and age differences in immune investment in a polygynous bat
Source: J Anim Ecol. 2026 Jun 30;95(8):1453–63. doi: 10.1111/1365-2656.70296 (PMC13439593; doi:10.1111/1365-2656.70296)
Supplement: Supplementary file 1 [file JANE-95-1453-s001.docx]

Supplement to

Leukocyte profiles reveal sex and age differences in immune investment in a polygynous bat

Gerald S. Wilkinson^1*^, Jillian A. Kaiser^1,2^, Katherine N. Armenta^1^, Ana S.G. Cunningham^1,3^, Severine B.S.W. Hex^1,4^, Alexis M. Lawson^1^, Jack G. Rayner^1^

^1^Department of Biology, University of Maryland, College Park, MD 20742 USA

^2^National Institute of Allergy and Infectious Diseases, National Institutes of Health, Bethesda, MD 20892 USA

^3^School of Medicine, University of North Carolina, Chapel Hill, NC 27599 USA

^4^School of Psychology, Center for Research in Animal Behavior, Exeter University, Exeter EX4 4QG UK

*Corresponding author, email: [wilkinso@umd.edu](mailto:wilkinso@umd.edu)

Table S1. Number of individual bats sampled for NLR and body condition by sex, season, site and year.

| **Sex** | **Season** | **Site** | **2011** | **2023** | **2024** | **2025** | **2026** | **Total** |
| --- | --- | --- | --- | --- | --- | --- | --- | --- |
| F | Mating | Caura | 0 | 10 | 0 | 0 | 2 | 12 |
| F | Mating | IH2 | 0 | 0 | 14 | 14 | 20 | 48 |
| F | Mating | Tam | 0 | 35 | 37 | 24 | 25 | 121 |
| F | Lactation | Caura | 0 | 7 | 0 | 0 | 0 | 7 |
| F | Lactation | IH2 | 0 | 18 | 0 | 0 | 0 | 18 |
| F | Lactation | Tam | 0 | 24 | 0 | 0 | 0 | 24 |
| F | Post-lactation | Guan | 37 | 0 | 0 | 0 | 0 | 37 |
| F | Post-lactation | Tam | 18 | 0 | 0 | 0 | 0 | 18 |
| M | Mating | Caura | 0 | 9 | 0 | 0 | 3 | 12 |
| M | Mating | IH2 | 0 | 0 | 10 | 18 | 17 | 45 |
| M | Mating | Tam | 0 | 19 | 13 | 10 | 10 | 52 |
| M | Lactation | Caura | 0 | 10 | 0 | 0 | 0 | 10 |
| M | Lactation | IH2 | 0 | 18 | 0 | 0 | 0 | 18 |
| M | Lactation | Tam | 0 | 14 | 0 | 0 | 0 | 14 |
| M | Post-lactation | Caura | 2 | 0 | 0 | 0 | 0 | 2 |
| M | Post-lactation | Guan | 58 | 0 | 0 | 0 | 0 | 58 |
| M | Post-lactation | Lop | 3 | 0 | 0 | 0 | 0 | 3 |
| M | Post-lactation | Tam | 12 | 0 | 0 | 0 | 0 | 12 |

Table S2. Number of individual bats sampled for cortisol by sex, season, site and year.

| **Sex** | **Season** | **Site** | **2023** | **2024** | **2025** | **2026** | **Total** |
| --- | --- | --- | --- | --- | --- | --- | --- |
| F | Lactation | IH2 | 12 | 0 | 0 | 0 | 12 |
| F | Lactation | Tam | 6 | 0 | 0 | 0 | 6 |
| F | Mating | Caura | 6 | 0 | 0 | 0 | 6 |
| F | Mating | IH2 | 0 | 5 | 6 | 6 | 17 |
| F | Mating | Tam | 22 | 8 | 0 | 0 | 30 |
| M | Lactation | Caura | 4 | 0 | 0 | 0 | 4 |
| M | Lactation | IH2 | 11 | 0 | 0 | 0 | 11 |
| M | Lactation | Tam | 8 | 0 | 0 | 0 | 8 |
| M | Mating | Caura | 8 | 0 | 0 | 0 | 8 |
| M | Mating | IH2 | 0 | 3 | 9 | 8 | 20 |
| M | Mating | Tam | 12 | 5 | 0 | 0 | 17 |

Table S3. Comparison of fit as measured by the corrected Akaike Information Criterion (AICc) of linear mixed models that predict ln(NLR) in 209 adult males (147 bachelor, 62 harem) using status, age and their interaction as fixed effects and individual and season as random effects.

| Model | AICc | ΔAICc |
| --- | --- | --- |
| Age | 492.17 | 0.00 |
| Age + Status | 493.27 | 1.10 |
| Age * Status | 494.00 | 1.83 |

Table S4. Comparison of fit as measured by the corrected Akaike Information Criterion (AICc) of linear mixed models that predict ln(NLR) using sex-specific relative body mass (condition), sex, age and interactions as fixed effects with individual and season as random effects (N = 494).

| Model | AICc | ΔAICc |
| --- | --- | --- |
| Sex + Age | 1088.19 | 0.00 |
| Condition + Sex + Age + Sex*Condition | 1098.82 | 10.63 |
| Condition + Sex + Age + Sex*Condition + Sex*Age | 1099.80 | 11.61 |
| Condition + Sex + Age + Age*Condition + Sex*Condition | 1100.90 | 12.71 |
| Condition + Sex + Age + Age*Sex | 1101.09 | 12.90 |
| Condition + Sex + Age | 1101.13 | 12.94 |
| Condition + Sex + Age + Sex*Condition + Sex*Age + Condition*Age | 1101.86 | 13.67 |
| Condition + Sex + Age + Age*Condition | 1102.03 | 13.84 |
| Condition*Sex*Age | 1103.96 | 15.77 |
| Condition + Sex + Sex*Condition | 1111.34 | 23.15 |

Figure S1. Relationship between age estimated by DNAm or mark recapture interval plotted against toothwear index for each sex. Least squared regression used to predict age for females is age = -2.0591 + 3.4318(TI), r^2^ = 0.652, N = 520 and for males it is age = -1.2719 + 2.5798 (TI), r^2^ = 0.604, N = 156.

Figure S2. Distribution of ages of wild female (F) and male (M) greater spear-nosed bats with estimates of NLR. The method of age estimation is indicated as DNA methylation clock (DNAm), recapture of a bat whose age had previously been estimated with the DNA methylation clock (DNAmR), recapture of a bat initially banded as a pup (Recap), or using a sex-specific regression derived from comparing known aged animals to their toothwear score (cf. Figure S1).

Figure S3. Neutrophil-to-lymphocyte ratio (lnNLR) for animals sampled during the mating season in more than one year with females in red and males in blue.

ln(NLR)

Figure S4. Plots of ln(cortisol corrected for specific gravity) and residual ln(cortisol corrected for specific gravity and collection hour) with median, quartiles and outliers by season with females in red and males in blue.

Figure S5. Plots showing median, quartiles and outliers for body condition (sex-specific residual body mass relative to forearm length) by season with females in red and males in blue.
